# Psychological distress among frontline workers during the COVID-19 pandemic: A mixed-methods study

**DOI:** 10.1371/journal.pone.0255510

**Published:** 2021-08-05

**Authors:** Wieke E. van der Goot, Robbert J. Duvivier, Nico W. Van Yperen, Marco A. de Carvalho-Filho, Kirsten E. Noot, Renee Ikink, Rijk O. B. Gans, Eveline Kloeze, Jaap E. Tulleken, A. J. Jolanda Lammers, A. Debbie C. Jaarsma, Wouter F. W. Bierman

**Affiliations:** 1 Martini Academy, Martini Hospital, Groningen, The Netherlands; 2 Center for Educational Development and Research in Health Sciences (CEDAR), Lifelong Learning, Education and Assessment Research Network (LEARN), University Medical Center Groningen, University of Groningen, Groningen, The Netherlands; 3 Parnassia Psychiatric Institute, The Hague, The Netherlands; 4 Department of Psychology, University of Groningen, Groningen, The Netherlands; 5 ICVS Life and Health Sciences Research Institute, School of Medicine, University of Minho, Braga, Portugal; 6 Department of Medicine, University Medical Center Groningen, University of Groningen, Groningen, The Netherlands; 7 Department of Critical Care, University Medical Center Groningen, University of Groningen, Groningen, The Netherlands; 8 Department of Internal Medicine / Infectious Diseases, Isala, Zwolle, The Netherlands; 9 Faculty of Veterinary Medicine, Utrecht University, Utrecht, The Netherlands; China University of Mining and Technology, CHINA

## Abstract

**Background:**

Novel virus outbreaks, such as the COVID-19 pandemic, may increase psychological distress among frontline workers. Psychological distress may lead to reduced performance, reduced employability or even burnout. In the present study, we assessed experienced psychological distress during the COVID-19 pandemic from a self-determination theory perspective.

**Methods:**

This mixed-methods study, with repeated measures, used surveys (quantitative data) combined with audio diaries (qualitative data) to assess work-related COVID-19 experiences, psychological need satisfaction and frustration, and psychological distress over time. Forty-six participants (nurses, junior doctors, and consultants) completed 259 surveys and shared 60 audio diaries. Surveys and audio diaries were analysed separately.

**Results:**

Quantitative results indicated that perceived psychological distress during COVID-19 was higher than pre-COVID-19 and fluctuated over time. Need frustration, specifically autonomy and competence, was positively associated with psychological distress, while need satisfaction, especially relatedness, was negatively associated with psychological distress. In the qualitative, thematic analysis, we observed that especially organisational logistics (rostering, work-life balance, and internal communication) frustrated autonomy, and unfamiliarity with COVID-19 frustrated competence. Despite many need frustrating experiences, a strong connection with colleagues and patients were important sources of relatedness support (i.e. need satisfaction) that seemed to mitigate psychological distress.

**Conclusion:**

The COVID-19 pandemic resulted in an increase of psychological distress among frontline workers. Both need frustration and need satisfaction explained unique variance of psychological distress, but seemed to originate from different sources. Challenging times require healthcare organisations to better support their professionals by tailored formal and informal support. We propose to address both indirect (e.g. organisation) and direct (e.g. colleagues) elements of the clinical and social environment in order to reduce need frustration and enhance need satisfaction.

## Introduction

In March 2020, the World Health Organisation declared the coronavirus disease 2019 (COVID-19) outbreak as a pandemic [1]. The COVID-19 pandemic has had an enormous impact on both healthcare systems and healthcare professionals [2]. Knowledge from previous virus outbreaks (e.g. SARS, MERS, and Ebola) indicated that healthcare professionals in contact with affected patients (so-called frontline workers) experienced more psychological distress and were more at risk for post-traumatic stress symptoms than healthcare professionals who had not been in contact with affected patients [3–6].

Based on previous research, there are strong indications that novel virus outbreaks may increase psychological distress among frontline workers during COVID-19. Emerging research on COVID-19 from several countries indicates that frontline workers report symptoms of depression, anxiety, and stress [7–9]. Reported risk factors included lack of perceived psychological preparedness and self-efficacy to help patients, social isolation, and fear of improper use or unavailability of personal protective equipment (PPE) and associated infection risks [10–12]. This is problematic, because psychological distress may lead to ill-being, which has detrimental effects on professionals, but also on healthcare organisations and patient safety [13]. The effect of COVID-19 on frontline workers’ experiences therefore requires further exploration. A better understanding of the challenges and changes in work dynamics of frontline workers during the COVID-19 pandemic may help to develop tailored interventions that effectively support frontline workers’ psychological health.

A useful lens to explore frontline workers’ experiences of psychological distress is self-determination theory (SDT). This theory on human motivation and socialisation distinguishes three basic psychological needs (i.e. autonomy, competence, and relatedness) that are essential for healthy development and growth [14, 15]. First, the need for autonomy describes actions for which people feel a full sense of choice and free will [15–17]. Second, the need for competence refers to a sense of effectiveness and capability to execute and fulfil tasks [17–19]. Finally, the need for relatedness refers to the desire to feel connected to others and to have a sense of belonging [17, 20]. Work environments that facilitate satisfaction of these basic psychological needs prompt positive psychological outcomes, such as enhanced performance and greater psychological well-being [21, 22]. Conversely, work environments that frustrate these needs can cause psychological distress and maladaptive functioning [23]. Need frustration is experienced when basic psychological needs are thwarted [24, 25]. For example, when one feels obliged to act, think, or feel a certain way (autonomy frustration), feels insecure or disappointed about their skills (competence frustration), or feels excluded from their team (relatedness frustration) [23].

Changing circumstances due to COVID-19, such as more shifts, new roles, and highly protocolled care, may impede psychological need satisfaction and may evoke psychological need frustration. Research indicates that need satisfaction and need frustration should be treated as different constructs [24, 26]. While need satisfaction is deemed essential for well-being, a lack of need satisfaction may result in suboptimal growth and development, whereas need frustration may decrease well-being and promote health issues [23, 27]. In this study, we assumed that novel virus outbreaks would affect frontline workers’ basic psychological need satisfaction and frustration. Specifically, due to the unpredictable course of the COVID-19 pandemic and (the associated) top-down decision-making processes to organise care, we expected a decrease in autonomy satisfaction and an increase in autonomy frustration. Furthermore, a lack of knowledge about COVID-19, its prognosis, and treatment options was likely to decrease frontline workers’ competence satisfaction and increase their competence frustration. Finally, we expected that the effects on frontline workers’ need for relatedness could go either way. In a pandemic, frontline workers typically work in teams especially assembled or altered for the situation. These newly composed COVID-19 teams may enhance frontline workers’ relatedness satisfaction, because frontline workers in these teams may create supportive peer networks among themselves. Alternatively, new and changing teams may result in less relatedness satisfaction and more relatedness frustration, as professionals may feel more disconnected from each other.

To better understand these anticipated effects of the COVID-19 pandemic on frontline workers’ psychological needs and psychological distress, we conducted a mixed-methods study–with both quantitative and qualitative measurements. Combining quantitative and qualitative data is important to gain more insight into frontline workers’ experiences and perceptions in a dynamic and complex work environment. Specifically, in our quantitative study, we predicted a positive relation between need frustration and psychological distress (*Hypothesis 1a)*, and a negative relation between need satisfaction and psychological distress (*Hypothesis 1b*). In addition, we predicted that among frontline workers, the level of perceived psychological distress during the COVID-19 pandemic was higher than pre-COVID-19 *(Hypothesis 2a)*, but that these levels would decrease when the number of COVID-19 infections declined (*Hypothesis 2b*). For our qualitative analysis, we used audio diaries to acquire frontline workers’ actual work-related experiences to enrich and illustrate our quantitative data. With these qualitative data, we explored the interplay of various elements of the work environment that may have supported or frustrated frontline workers’ basic psychological needs, and accordingly, affected their psychological distress.

## Methods

### Design and procedure

We adopted an explorative longitudinal design with both repeated surveys (quantitative data) and audio diaries (qualitative data). Data were collected at six time points (T1, T2, T3, T4, T5, and T6) from April to November 2020. Frontline workers from two hospitals (one university medical centre and one general teaching hospital) in the Netherlands were recruited for participation in this study. Inclusion criteria were met if the participant was a consultant, junior doctor, or nurse, and worked on a ward or intensive care unit that provided care for COVID-19 patients. From April to May, participants were recruited via e-mail and by flyers on the wards by WEvdG, WFWB, EK, JET, and AJJL. If they were willing to participate, they contacted WEvdG and/or followed a web link to the first survey. All participants received an information letter about the study. An informed consent form preceded the first survey, in which participants indicated whether they would participate in the surveys only or both the surveys and audio diaries. After this survey, participants filled out a contact form for follow-up surveys and/or the audio diaries. The survey was designed by WEvdG, RJD, NWVY, ADCJ, and WFWB. The measures reported in this paper are part of this survey. Please refer to the supporting information for the survey items (S1 File).

The Ethical Review Board of the University Medical Center Groningen gave approval for this study, because it did not fall within the scope of the Medical Research Involving Human Subjects Act (METc 2020/186).

### Surveys

#### Quantitative data collection & sample

Quantitative data were collected at all six time points (T1 –T6). Between April and June, participants received four surveys (T1 –T4) with an interval of seven days between each survey. In this period, regular, non-COVID care was largely downscaled in the participating hospitals. After the fourth survey, all participants received a digital voucher (€10) for their participation. In August, participants received the fifth survey (T5). At this time, non-COVID hospital care had mostly upscaled again. In October, participants received the sixth and final survey (T6). Around this time, the number of hospitalised COVID-19 patients was progressively increasing, accompanied by partial downscaling of non-COVID care.

The study population (N = 46) consisted of eight consultants (17.4%), 15 junior doctors (32.6%), and 23 nurses (50.0%). Participants were on average 35.4 years old (SD = 9.8), and 78.3% were female (n = 36). One participant withdrew their participation after T1. Thirty-seven participants completed all six surveys (T1 –T6), four participants completed five surveys, and four participants completed four surveys. Forty participants completed the last survey (T6). In total, we received 259 surveys, resulting in 6.16% (17/276) missing data. An additional number of 14 (5.41%) surveys did not include the basic need satisfaction and frustration scale, because this scale was only assessed if the participant had worked during the previous week (i.e. some participants were off-duty during one of the measurements). The completion rate was 88.8% (245 out of 276 surveys).

#### Quantitative measures

*Demographic variables*. We collected the following demographic characteristics of participants: age, sex, professional occupation, department where they normally worked in non-COVID times, and average hours worked per week regularly.

*Need satisfaction & frustration*. Work-related autonomy, competence, and relatedness satisfaction and frustration were measured six times with the diary version of the BPNS/F scale [26, 28, 29]. The response scale ranged from 1 (*not true at all*) to 5 (*completely true*). Example items included: “Last week, I felt a sense of choice and freedom in my job” (autonomy satisfaction; aggregated α = .63, with a range between α = .34 to α = .85), “Last week, I felt confident that I could do things well” (competence satisfaction; aggregated α = .83, with a range between α = .28 to α = .93), “Last week, I felt connected with people who care for me, and for whom I care” (relatedness satisfaction; aggregated α = .84, with a range between α = .77 to α = .90), “Last week, most of the things I did felt like ‘I had to’” (autonomy frustration; aggregated α = .73, with a range between α = .60 to α = .83), “Last week, I felt disappointed with many of my performances” (competence frustration; aggregated α = .73, with a range between α = .54 to α = .83), and “Last week, I felt excluded from my team” (relatedness frustration; aggregated α = .78, with a range between α = .72 to α = .85). Higher scores indicated more satisfaction or frustration of work-related needs, respectively.

*Psychological distress*. We assessed participants’ perceived psychological distress with the 12-item version of the General Health Questionnaire (GHQ) [30]. We asked participants to rate their perceived psychological distress over the last seven days compared to pre-COVID-19 (before March 2020). Example items from the GHQ-12 are “Have you been feeling reasonably happy over the past seven days, all things considered” (1 = happier than before; 2 = as happy as before; 3 = less happy than before; 4 = much less happy than before) and “Have you felt that you were playing a useful part in things over the past seven days” (1 = more useful than before; 2 = just as useful as before; 3 = less useful than before; 4 = much less useful than before). Participants rated all items on a four-option multiple-choice scale. We used the average GHQ-12 Likert score for statistical analysis and the bimodal scoring method (0-0-1-1, range 0–12), to screen for psychological distress among participants, with a threshold of 1 / 2. Higher scores indicated more psychological distress than before the COVID-19 pandemic. A bimodal score > 1 indicated that participants experienced somewhat more psychological distress than before COVID-19. A bimodal score > 2 indicated that the participant experienced more severe psychological distress than before the COVID-19 pandemic, which may lead to mental health disorders, and might be considered for follow-up mental health testing [31]. The reliability estimates ranged between α = .65 and α = .82, with an aggregated average of α = .76.

#### Quantitative data analysis

Descriptive data analysis was performed with SPSS version 26. We used MLwiN version 3.05 [32] to employ multilevel analyses.

### Audio diaries

#### Qualitative data collection & sample

The qualitative data (audio diaries) were collected during the first four time points (T1 –T4). The participants were instructed to record 1 to 3 audio diaries per week over a period of four weeks (see S2 File). Fifteen participants (ten nurses, two consultants, and three junior doctors) shared 60 audio diaries, 13 of them were female (86.7%).

#### Qualitative measures

*Audio diaries*. To explore participants’ recent work experiences during the COVID-19 pandemic, we asked participants to record experiences that had a significant impact on them, either positive or negative (see S2 File for all prompts). All shared entries were in Dutch, with a duration of one to eight minutes, and were transcribed verbatim before analysis.

#### Thematic analysis

We used a constructivist perspective to analyse the qualitative data, assuming that each participant shared their perspective on reality [33]. We used the six phases of thematic analysis to structure our analysis [34] (see S3 File for additional information). Data analysis started with familiarisation with the data by reading and re-reading all of the transcripts. Second, the transcripts were open-coded. Third, we developed a deductive thematic framework with autonomy, competence, and relatedness satisfaction and frustration, respectively. Fourth, we used the thematic framework for deductive analysis and discussed and reviewed the themes. Fifth, we (re)defined and interpreted the themes, leading to the results section in this paper. Finally, we wrote the draft paper and triangulated the qualitative and quantitative data. We used Atlas.ti software version 8 (ATLAS.ti Scientific Software Development GmbH, Berlin, Germany) to support data organisation and management.

## Results

### Quantitative data

Table 1 presents the means, standard deviations, and correlations between the study variables. In line with Hypothesis 1, psychological distress was negatively associated with need satisfaction (*p* < .001), and positively associated with need frustration (*p* < .001).

**Table 1:** Means, standard deviations, and correlations between the study variables.

Variable		M	SD	1	2	3	4	5	6
**1**	**Autonomy satisfaction**	3.48	0.67	–					
**2**	**Competence satisfaction**	4.02	0.57	.24**	–				
**3**	**Relatedness satisfaction**	3.86	0.72	.18**	.25**	–			
**4**	**Autonomy frustration**	2.13	0.73	-.48**	-.11	-.22**	–		
**5**	**Competence frustration**	1.93	0.68	-.33**	-.36**	-.22**	.27**	–	
**6**	**Relatedness frustration**	1.62	0.56	-.23**	-.18**	-.57**	.26**	.24**	–
**7**	**Psychological distress**	1.80	0.30	-.25**	-.19**	-.26**	.32**	.36**	.27**

*p < .05

**p < .001. M = mean, SD = standard deviation.

#### Multilevel analyses

Our data were hierarchically structured, with six repeated measures (T1 –T6, Level 1) nested in participants (Level 2). We created seven null models (see S1 Table), one for each variable, to examine the distribution of variance at the within- (Level 1) and between-person level (Level 2). In all null models, the within-person variance was larger than the between-person variance. Given these significant differences, multilevel analysis was appropriate to test our hypotheses.

#### Hypothesis testing

Our first hypothesis predicted a positive relation between need frustration and psychological distress (*Hypothesis 1a)*, and a negative relation between need satisfaction and psychological distress (*Hypothesis 1b)*. First, we tested the variance in psychological distress that was explained by need frustration (Table 2, Model 1). Multilevel analysis showed that need frustration explained 9.0% variance at the between-person level and 9.0% variance at the within-person level. This model provided a significantly better fit than the null model (χ^2^_change null model_ (3) = 45.81; *p* < .001). We observed a significant increase in psychological distress for autonomy frustration (*p* = .005), competence frustration (*p* < .001), and relatedness frustration (*p* = .008).

**Table 2:** Summary of the model estimates for psychological distress.

Parameter	Model 1				Model 2				Model 3			
	b	SE	t(42)	*p*	b	SE	t(42)	*p*	b	SE	t(39)	*p*
**Intercept**	1.80	0.02	75.08	< .001	1.80	0.03	66.70	< .001	1.80	0.03	72.04	< .001
**Autonomy frustration**	0.07	0.02	2.96	.005					0.06	0.03	2.27	.029
**Competence frustration**	0.13	0.03	4.68	< .001					0.12	0.03	3.97	< .001
**Relatedness frustration**	0.09	0.03	2.67	.011					0.04	0.04	0.92	.363
**Autonomy satisfaction**					-0.08	0.03	-2.82	.007	-0.02	0.03	-0.63	.530
**Competence satisfaction**					-0.05	0.03	-1.74	.089	-0.03	0.03	-1.00	.323
**Relatedness satisfaction**					-0.10	0.03	-3.85	< .001	-0.07	0.03	-2.33	.025
**Explained variance (%)**												
**Between-person level**	8.99				1.12				5.62			
**Within-person level**	8.99				7.87				12.36			
2*log likelihood	34.01				49.14				26.29			
**χ**^**2**^_**change null model**_ **(3)**	45.81			< .001	30.68			< .001	53.54			< .001
**χ**^**2**^_**change 1**_ **(3)**									7.73			.052
**χ**^**2**^_**change 2**_ **(3)**									22.85			< .001

b = unstandardised coefficient, SE = standard error, t(df) = t-test statistic with degrees of freedom within brackets, *p* = p-value.

Second, we tested the variance in psychological distress that was explained by need satisfaction (Table 2, Model 2). Multilevel analysis showed that need satisfaction explained 1.1% variance at the between-person level, and 7.9% variance at the within-person level. This model provided a significantly better fit than the null model (χ^2^_change null model_ (3) = 30.69, *p* < .001). We observed a significant decrease in psychological distress for autonomy satisfaction (*p* = .007), and relatedness satisfaction (*p* < .001), but not for competence satisfaction (*p* = .089).

Third, we combined Model 1 and Model 2 to assess if frustrated and satisfied needs explained additional variance in psychological distress (Table 2, Model 3). This model explained 5.6% variance of psychological distress at the between-person level, and 12.4% at the within-person level. Model 3 provided a significantly better fit than the need satisfaction only model (χ^2^_change 2_ (3) = 22.85; *p* < .001), and the need frustration only model (χ^2^_change 1_ (3) = 7.73; *p* = .052). We observed a significant decrease in psychological distress for relatedness satisfaction (*p* = .025), but not for competence satisfaction (*p* = .323), or autonomy satisfaction (*p* = .530). We observed a significant increase in psychological distress for autonomy frustration (*p* = .029), and competence frustration (*p* < .001), but not for relatedness frustration (*p* = .363). Hence, we conclude that Hypothesis 1 was only partly supported: unique variance of psychological distress was explained by autonomy and competence frustration (*Hypothesis 1a)*, and by relatedness satisfaction (*Hypothesis 1b*).

In our second hypothesis, we predicted that the level of perceived psychological distress among frontline workers was higher than before the COVID-19 pandemic *(Hypothesis 2a)*, but that these levels would decrease over time when COVID-19 infection rates declined *(Hypothesis 2b)*. As indicated, we asked the participants to rate their perceived psychological distress over the last seven days *compared to pre-COVID-19 (i*.*e*. *before March 2020)*. To test the second hypothesis, we first calculated perceived psychological distress among frontline workers and used the cut-off scores of Goldberg et al. [31]. Table 3 shows that relative to pre-COVID-19, a substantial percentage (27.9% - 50.0%) of the participants experienced more psychological distress across different time points, which supports *Hypothesis 2a*. More specifically, 7.0% - 20.0% experienced somewhat more psychological distress (score > 1–2), and 13.3–30.0% experienced more severe psychological distress (score > 2) during COVID-19. Mann-Whitney tests indicated that, across all six time points, the group of participants that experienced more psychological distress (GHQ > 1) had a significantly higher median score (*p* < .001) than the group of participants that did not experience more psychological stress (GHQ ≤ 1) than before COVID-19 (see S2 Table).

**Table 3:** Perceived psychological distress across time points.

Measurement	N	Likert M (SD)	Bimodal M (SD)	Range	GHQ-score > 1–2	GHQ-sore > 2
T1	46	1.85 (.33)	1.87 (2.28)	0–9	6 (13.0%)	13 (28.3%)
T2	45	1.76 (.25)	1.11 (1.51)	0–7	7 (15.6%)	6 (13.3%)
T3	43	1.78 (.27)	1.14 (1.70)	0–7	7 (16.3%)	7 (16.3%)
T4	43	1.75 (.27)	1.00 (1.61)	0–7	3 (7.0%)	9 (20.9%)
T5	42	1.81 (.34)	1.31 (2.07)	0–10	6 (14.3%)	8 (19.1%)
T6	40	1.86 (.34)	1.85 (1.99)	0–9	8 (20.0%)	12 (30.0%)

N = number of participants, M = mean, SD = standard deviation.

Note that Table 3 also shows that psychological distress fluctuated over time. We tested these fluctuations (*Hypothesis 2b)*, with multilevel analysis (see S3 Table). The results indicated a trend that perceived psychological distress was somewhat lower on T2 (p = .081) and T4 (p = .050) compared to T1. This model did not explain more variance than the null model with psychological distress only (χ^2^_change null model_ (5) = 7.52; *p* = .185). Hence, we conclude that *Hypothesis 2* was partially supported. A substantial number of participants perceived more psychological distress during COVID-19 than before, but we found no statistically significant changes in psychological distress over time.

### Audio diaries

Our thematic framework included six themes to categorise and describe the recordings, namely ‘autonomy support’, ‘competence support’, ‘relatedness support’, ‘autonomy frustration’, ‘competence frustration’, and ‘relatedness frustration’. Nearly all experiences illustrated an interplay between different needs. Therefore, we divided the results into need supportive and need frustrating experiences. Relatedness support was predominantly described in the need supportive experiences, while autonomy frustration and competence frustration were especially illustrated in the need frustrating experiences. In addition, the actors in need supportive and frustrating experiences differed. Need supportive experiences often included specific people (e.g. colleagues, patients, family members). Need frustrating experiences, however, often included COVID-19 itself, or groups as actors or systems (e.g. organisations, teams, or society).

#### Need frustrating experiences

In many need frustrating experiences, the unpredictable course of COVID-19 or organisational logistics played a role. In particular, the need for autonomy was frustrated, illustrated by participants who felt unable to support patients or organise their work effectively. Limited experience with COVID-19 (competence frustration) and frequently changing teams (relatedness frustration) further frustrated their needs. The use of personal protective equipment (PPE) complicated collaboration between colleagues or communication with patients and family members. Organisational logistics (e.g. work schedules, rules and regulations, communication) led participants to experience little control and influence over their work and work-life balance (autonomy frustration).

*COVID-19 care*. The unpredictable course of COVID-19 had an enormous impact on many participants. Participants perceived limited possibilities to effectively support patients and often felt defeated when patients deteriorated quickly despite the care they provided. Multiple participants experienced that ‘fate’ predicted the course of the disease and they could not (entirely) rely on their clinical skills and experience due to the absence of a concise treatment.

*“So*, *in that sense, it is a constantly unpredictable situation and that, yes, sometimes that causes me to be extra worried for patients while I would normally not be for such patients. […] Anyway, I now find it scary, and now I notice that I-, um, for example, last night I was awake, and I started thinking whether I really did everything right. Whether I should have done extra checks.” (P3)*

These worries increased when participants worked in frequently changing teams or when teams were stressed or frustrated. This hampered the connection and trust among colleagues. Some participants reflected that they needed some time to feel comfortable in a new role and new team. Others experienced more difficulty relying on the skills of colleagues they did not know, which impeded their feelings of trust.

Another example from a nurse illustrated how they felt helpless to comfort a patient who just returned from the ICU to the COVID-ward. This patient woke up panicked, and started to hyperventilate. Despite all their efforts, the doctors and nurses were unable to comfort the patient and take their shortness of breath and anxiety away. The nurse reflected afterwards: *“I cried when I got home*, *just because you só badly want someone to–to be comfortable and to help them–and not being able to help someone*, *that you only can just stand by them*, *that makes me feel helpless*. *(P43)*.

This feeling of inability to comfort patients was strengthened by the use of PPE, which impeded contact and communication with patients, but also made daily care more tiring than normal: “*Because they also only see*, *uh*, *a blue suit and a mask and a hairnet and they do not see the person behind that*, *so it is harder to reassure [patients] than normally*.” (P2). On top of that, the obligatory use of PPE and strict rules to prevent the spreading of the disease impeded participants to lend a helping hand or support colleagues. This was especially hard during busy shifts, where participants saw their colleagues struggle to manage their work while they could only watch.

*Organisational logistics*. Organisational logistics frustrated participants’ needs in different ways. We found two categories related to communication that impeded need support, namely experiences associated with visiting arrangements and experiences associated with changing rules and regulations for the staff.

First, the strict visiting arrangements frustrated participants’ autonomy and competence. Many participants shared experiences where they needed to bring bad news. Normally, family members would visit the hospital for this and get the opportunity to say goodbye to their loved ones. During COVID-19, many participants had to bring bad news to family members under time pressure–because the patient deteriorated quickly and needed to be transferred to the ICU–and by telephone–because of the strict visiting arrangements: “*It is very difficult to give an emotional response [by telephone]*, *uh*, *such as putting an arm around them […] or looking them in the eyes to check how they handle this news*.*”* (P6) This challenged their perceived professional effectiveness of communication and provision of emotional support. In addition, visiting arrangements were changed frequently, but this was not always effectively communicated. This led to miscommunication and frustration within teams. For example, a pilot period to allow some visitors on the COVID-wards was not clearly communicated with the nurses, which resulted in fewer visitors than were possible and confusion within the team.

Second, limited involvement of the staff in organisational logistics especially frustrated participants’ autonomy. Many participants felt insufficiently involved in decision-making or incompletely informed by the organisation. Regulations from the hospital organisation led to (some degree of) uncertainty, unrest, or frustration for many participants. For example, it was unclear if or when participants would return to their own specialty to provide care for non-COVID patients. Some participants felt uncomfortable, useless or bored during quiet COVID-shifts with very few hospitalised patients. Multiple narratives included participants not being able to influence scheduling and rosters being available only last minute, in combination with frequently working on different wards with different teams. Some participants described a feeling of being ‘toyed with’:

*“For me personally*, *I-*, *it also exhausts me that you just don’t know where you stand*. *Especially if you have a family with children at home*, *a husband who in fact also has to work*, *and everyone has to adapt to my schedule ad hoc*.*”* (P6)

When miscommunications occurred about such regulations, nurses and junior doctors experienced no choice but to accept it because they stood lower in the overall hierarchy. In one case, participants heard that they needed to take care of more patients in the near future. This resulted in doubts about the future quality of care and impaired work satisfaction of nurses:

*“Uh*, *we have heard that we need to care for uh many more patients uh in total*. *Instead of uh 30 patients*, *a total of 60 approximately*. *[*…*] Uh*, *and there is unrest about that*. *We are concerned that the quality will decrease*, *that we will have less work satisfaction*, *uh*, *that- that care will be compromised and that this is insufficiently acknowledged*.*”* (P29)

#### Need supportive experiences

In many need supportive experiences, a feeling of connectedness with colleagues, patients, or family members played a central role. It seemed that the need for relatedness was vital to cope with emotionally demanding situations, such as deteriorating patients or the changes and uncertainties that were caused by COVID-19. Relatedness satisfaction seemed to promote competence and autonomy in the workplace, possibly due to an open and positive atmosphere within teams. Examples of autonomy support were more implicit and often related to a sense of competence. For example, knowledge and practical experience of dealing with COVID-19 (competence satisfaction) stimulated participants to organise care for patients in a way they felt was best (autonomy satisfaction).

*Interaction with colleagues*. Most participants felt connected with their colleagues. The uncertainties and challenges of working on COVID-wards promoted a sense of camaraderie, e.g. “*You have to do it as a team*, *and that’s the strength of uh working on wards like these"* (P33). This supported participants in coping with the emotionally demanding care that they had to provide and helped them share successes with their colleagues.

*"Yes*, *I sometimes find it difficult, to then- you are also in a kind of social isolation, and well, I am happy to see my colleagues. Luckily, I get a lot of support from my colleagues, and we were also able to talk about it well. Be able to evaluate afterward how intense it is.” (P1)*

Although some participants, at first, felt incompetent to care for COVID-19 patients, their confidence was built through training, using protocols, and open consultation with colleagues. There seemed to be an increased level of equality among colleagues because everyone at times struggled with the unpredictable course or treatment of COVID-19. An open atmosphere and a strong team spirit made it easier to ask colleagues for advice, build trust in each other, and address areas of improvement in daily care. Moreover, participants felt free to share their expertise with colleagues from a different professional background.

*“And I think that because of that, um, yes*, *people also feel more valued, because you’re not only doing regular COVID care, but you’re also be- being called upon your expertise from your own profession” (P3)*

*Interaction with patients & family members*. After a couple of weeks of working on a COVID-ward, participants felt increasingly confident in taking care of COVID-patients. Gaining more experience helped them to better explain to patients and family members what might happen during the disease, despite its unpredictability. Many participants shared that they felt closely connected to their patients, for example, through the in-depth conversations they had with patients. This strengthened their ability to emotionally or mentally support patients. Participants felt the freedom to take the time to talk to patients, because they cared for a limited number of patients each day:

*“Uh*, *it was uh, nice to support a patient on a social level and to have a conversation about that. And I really hope I’ve helped him. Uh, and what makes my profession so valuable–it’s uh–you’ve not become a physician to just prescribe pills, but you’re in a position to talk to people and uh try to find answers to their existential questions in life together.” (P6)*

The strong bond that participants felt with their patients seemed to stimulate autonomous behaviour. Participants used visiting policies, although they were strict and not always clearly communicated, to arrange short moments of contact between patients and their family members, or organised that patients were transferred to (rehabilitation) clinics near their families. They also used technical innovations, e.g. video calling, to establish valuable contact between patients and their families. As a participant (P6) stated, *“Uh*, *it was kind-of an eye-opener for me*, *at first I was hesitant because you think ‘will this work for effective communication*?*’*. *But actually*, *it was uh a nice method to communicate*.*”* In addition, another participant (P29) explained: “*Uh*, *it’s special to see that uh*, *loved ones can share some time through video-calling*. *But it’s also difficult to see them so far apart*, *in completely different worlds*.”

## Discussion

In this study, we quantitatively assessed frustration and satisfaction of basic psychological needs, and psychological distress during COVID-19, and qualitatively explored experiences of frontline workers. Our results provide insights about different elements of the social environment that affected psychological distress among frontline workers. While frontline workers experienced need frustration and increased levels of psychological distress, relatedness support seemed to help them deal with the challenges of the COVID-19 pandemic. In many narratives, we observed an interconnectedness between needs. In the next paragraphs, we discuss both quantitative and qualitative findings, their meaning from a theoretical and empirical perspective, and the implications for practice.

### Connection between qualitative and quantitative findings

The connection between quantitative and qualitative data yielded some interesting findings. COVID-19 resulted in an increase in perceived psychological distress compared to pre-COVID-19. Over time, 7.0% - 20.0% of participants experienced somewhat more psychological distress, and 13.3% - 30.0% of the participants experienced more severe levels of psychological distress. This is problematic, because these workers are at risk for mental health disorders [30]. Although 50.0–72.1% of our participants did not experience more psychological distress compared to pre-COVID-19, we need to interpret this finding with caution because we have no baseline pre-COVID-19 distress level of our sample. These findings do not indicate an absence of psychological distress pre-COVID-19. The narratives described many need frustrating experiences and illustrated that the emotional intensity of the work and a decrease in autonomy in their work impeded frontline workers’ psychological health during COVID-19. Therefore, the COVID-19 pandemic may put additional strain on healthcare staff, who are already at risk of distress, ill-being or burnout [35–37]. Fluctuations in perceived psychological distress of our sample seemed related to infection rates. This indicates that subsequent COVID-19 waves pose a challenge for frontline workers’ psychological health, despite increasing familiarity with the disease.

Work-related autonomy and competence frustration were associated with higher levels of psychological distress, while work-related relatedness satisfaction was associated with lower levels of psychological distress. The qualitative audio diaries endorsed and strengthened these findings. However, the quantitative levels of autonomy and competence frustration were relatively low. One explanation may be that qualitative and quantitative measures tap into different experiences. The surveys focussed on overall frustration or satisfaction of work-related needs over the past seven days, while the narratives focussed on specific work-related incidents that provoked emotional responses. The narratives illustrate that especially the need for autonomy was frustrated during COVID-19, frequently in close connection with competence frustration. Competence frustration was often related to unfamiliarity with COVID-19. Through increased knowledge about and experience with COVID-19, frontline workers regained a sense of control and self-efficacy in their work. Nevertheless, contagiousness of COVID-19 and all precautions (e.g. PPE) that they needed to take impeded them to organise their work effectively. Frontline workers’ autonomy seemed frustrated most by organisational logistics. Frequent and last-minute changes in work schedules and unclear communication within the organisation limited their possibilities to manage their work-life balance, and decreased the stability within teams. Both the quantitative and qualitative data clearly suggest that support of relatedness is valuable to reduce perceived psychological distress among frontline workers. Relatedness support, especially by colleagues, seemed vital to cope with emotionally demanding situations or work-related challenges during COVID-19. Often, relatedness support seemed to act in a reciprocal way. Frontline workers in our sample also supported relatedness of their colleagues’ relatedness (e.g. provide help, share experiences), patients, or family members (e.g. take time to connect and talk).

### Comparison with theoretical literature

Our findings illustrate that both need satisfaction and need frustration explained unique variance in psychological distress, specifically autonomy and competence frustration, and relatedness satisfaction. The effects of relatedness frustration, and both autonomy and competence satisfaction were less clear in our quantitative data, but our qualitative data suggest that these variables may also play a role in explaining psychological distress in our sample, although less obvious. Our findings add to the growing body of SDT literature that also takes the role of need frustration into consideration [23, 24, 27]. Central to SDT is that the social environment plays an essential role in need satisfaction or frustration [14, 15]. Our qualitative data suggest that an explicit focus on different elements of the social environment may help to explain how need frustration and need support simultaneously affect psychological distress (i.e. ill-being) or good mental health (i.e. well-being). We noticed in the narratives that different elements of the social (work) environment affected different needs. We distinguished the direct social environment (e.g. colleagues and patients), and the indirect social environment (e.g. the organisation). While the direct social environment seemed to support relatedness, the indirect elements of the social environment often frustrated autonomy and competence. Based on our findings, we pose that both direct and indirect elements of the social environment need to be addressed in complex and dynamic naturalistic settings, because need frustration and need satisfaction may originate from various sources simultaneously.

### Comparison with empirical studies

An important contribution of our findings to recent empirical studies is the combination of qualitative and quantitative data assessed multiple times, while many recent studies are cross-sectional and quantitative in nature [3, 5]. Many studies show an increase in psychological distress among healthcare staff during this pandemic [9, 11, 12, 38, 39]. Our findings are in line with literature that healthcare organisations can implement a diverse range of interventions [40] to support their staff during a pandemic, for example, through clear communication, sufficient training, rostering and support networks [5, 6]. Also, social support among colleagues is important to mitigate psychological distress [5]. We have captured the actual experiences of frontline workers, which indicate that both supportive and frustrating elements of the work environment simultaneously affect their perceived psychological distress. Our findings resonate with recent reviews [6, 40] that suggest that an institutional approach seems vital to mitigate psychological distress.

### Implications for practice

Psychological distress among frontline workers is problematic and requires attention from healthcare organisations to reduce the burden on the clinical staff during a pandemic [6]. We suggest some implications for practice that may help organisations to better support their professionals. However, future research is needed to assess the effectivity of specific interventions to support frontline workers [41, 42]. Participants’ narratives illustrate that working in the frontline was emotionally demanding, but sharing experiences and close connections with colleagues seemed to mitigate psychological distress among frontline workers. Therefore, we recommend that professionals be given protected time, during working hours, to reflect on and recover from the emotional impact of their work. Organisations could develop and strengthen internal supportive networks (e.g. peer support, psychological counselling or a hotline for professionals) that are easily accessible during and after working hours. This may support professionals to share and ventilate their experiences, to mitigate psychological distress. In addition, informal support (e.g. among colleagues) should be stimulated and organised (e.g. moments to allow quiet talk or activities to support connection among colleagues). Professionals should feel that ‘it is okay not to be okay’, and that emotional support and help are legitimate needs. Professionals requiring additional support (e.g. psychological, educational) should be stimulated to make use of tailored interventions to support their needs.

In addition, our findings illustrate that need support is frequently impeded and need frustration is evoked. To better support professionals’ autonomy, organisations need to become more flexible and responsive to their staff. Most importantly, the crisis management team(s) should actively collect and be responsive to feedback from professionals regarding the management of the crisis, work schedules (e.g. shifts, hours, professional roles), workload, work-home balance, and emotional demands of the crisis. We suggest creating stability within teams and providing flexible training and education when new insights arise in treatment and for new team members joining the team later on. This helps to promote professionals’ competence and to foster reciprocal relationships and belongingness within teams. Especially in an intermittent crisis, where successive waves of increased infection rates alternate with catching up with regular care, it is vital that organisations provide all the support they can to professionals to keep their work balanced and keep them psychologically healthy and engaged. These changes should be developed, evaluated and incorporated continuously to make healthcare organisations ready to act, not only for the next COVID-19 wave, but also for future challenges and healthcare crises that may severely impede professionals’ well-being.

### Strengths & limitations

Early in the pandemic, we were able to collect experiences from frontline workers in a time-efficient manner. With audio diaries, participants could spontaneously record and share work-related experiences with their own devices. These recordings were not guided to fit a particular perspective and were recorded shortly after the experience, thereby preventing recall bias [43, 44]. A drawback of this method was that we could not further explore or follow up on these shared entries. Nevertheless, in combination with the repeated surveys, we were able to triangulate the data to expand our exploration of the social work environment that affected frontline workers’ basic psychological needs and psychological distress [45].

We used SDT’s basic psychological needs for both our quantitative and qualitative analysis. This provided valuable insight into frontline workers’ experiences. To prevent a too narrow thematic focus by our theory-driven qualitative analysis, we started with an inductive analysis. Through frequent team discussions and memo writing, we created a thorough overview of the dataset. The subsequent deductive thematic analysis provided a focussed description of the basic needs, wherein the role of the work environment was taken into consideration. Moreover, the research team included diverse professional backgrounds. This promoted reflexivity to interpret the findings from a theoretical perspective as well as for clinical practice. While a different theoretical lens or analytical approach may have resulted in different findings, we believe that the results represent the data in a theoretically and practically valuable way.

Our relatively small quantitative sample size and nurses’ overrepresentation in our audio diaries limit the generalisability and transferability of our findings. The reliabilities of our need satisfaction and frustration scales were poor for autonomy and competence support. Moreover, a full multilevel analysis requires a larger sample size. While our qualitative dataset was sufficient for thematic analysis, these results were mainly based on nurses’ and, to a somewhat lesser extent, junior doctors’ experiences, but did not seem to encapsulate consultants’ experiences. Therefore, our results remain exploratory in nature. Future research is needed to assess the unique value of each predictor variable on psychological distress.

## Conclusion

Our study illustrates that both psychological need frustration as well as psychological need satisfaction are needed to effectively investigate frontline workers’ psychological distress. Different methods shed additional light on the elements of the direct and indirect social environment that influence need satisfaction and need frustration of people in complex and dynamic naturalistic settings. Challenging times, such as COVID-19, require healthcare organisations to better support their professionals by tailored formal and informal support networks.

## Supporting information

S1 File Survey items. (DOCX)

S2 File Audio diary instruction. Instructions and prompts for participants to record and share their entries.
(DOCX)

S3 File Thematic analysis. The six steps of thematic analysis and the reflexivity paragraph.
(DOCX)

S1 Table The distribution of between- and within-person variance for the predictor variables (null models). (DOCX)

S2 Table Non-parametric test for differences in psychological distress. (DOCX)

S3 Table Model with fluctuations in psychological distress over time. (DOCX)
