# Supplementary material for: Psychological distress among frontline workers during the COVID-19 pandemic: A mixed-methods study
Source: PLoS One. 2021 Aug 5;16(8):e0255510. doi: 10.1371/journal.pone.0255510 (PMC8341539; doi:10.1371/journal.pone.0255510)
Supplement: S1 File — (DOCX) [file pone.0255510.s001.docx]

## **S1 File. Survey items.**

### **Demographic data**

1. What is your age? (open question)
2. What is your sex?
   1. Male
   2. Female
3. What is your professional role?
   1. Consultant
   2. Junior doctor
   3. Nurse
   4. Other, namely…
4. In which specialty are you normally employed? (open question)
5. How many hours do you work normally per week?
   1. 0 – 12
   2. 13 – 24
   3. 25 – 36
   4. 37 – 48
   5. > 48

### **Work situation in the past 7 days (during COVID-19)**

*The following questions are about your work and job situation of the past 7 days.*

1. Did you work in a department where COVID patients are admitted?
   1. Yes, namely
      1. COVID-Intensive care unit
      2. COVID-ward
   2. No, I was working at another department
   3. No, I was off-duty the past week
2. How many hours did you work in the past 7 days?
   1. 0 – 12
   2. 13 – 24
   3. 25 – 36
   4. 37 – 48
   5. > 48
3. Have you attended any COVID-19 training in the past 7 days?
   1. No
   2. Yes, namely...
4. What type of shifts have you worked in the past 7 days? (multiple answer options)
   1. Day shift
   2. Evening shift
   3. Night shift
   4. Weekend shift
   5. Other, namely…

### **Basic Psychological Need Satisfaction and Frustration – Diary Version**

*We would like to ask you to think back to the past 7 days. Which feelings best describe the experiences you have had at work in the past 7 days? You can give each item a score from 1 ('not true at all') to 5 ('completely true') to indicate to what extent a certain feeling applies to you at this moment. By 'team' we mean your colleagues in the workplace (e.g. consultants, junior doctors, nurses, supporting staff).*

Last week at work…

| 1. …I felt a sense of choice and freedom in my job. | 1 | 2 | 3 | 4 | 5 |
| --- | --- | --- | --- | --- | --- |
| 1. …I felt disappointed with many of my performances. | 1 | 2 | 3 | 4 | 5 |
| 1. …I felt that people who are important to me were cold and distant towards me. | 1 | 2 | 3 | 4 | 5 |
| 1. …most of the things I did felt like “I had to”. | 1 | 2 | 3 | 4 | 5 |
| 1. …I felt confident that I could do things well. | 1 | 2 | 3 | 4 | 5 |
| 1. …I felt that my decisions reflected what I really wanted. | 1 | 2 | 3 | 4 | 5 |
| 1. …I felt connected with people who care for me, and for whom I care. | 1 | 2 | 3 | 4 | 5 |
| 1. …I felt excluded from my team. | 1 | 2 | 3 | 4 | 5 |
| 1. …I felt forced to do many things I didn’t choose to do. | 1 | 2 | 3 | 4 | 5 |
| 1. …I felt capable of doing my job. | 1 | 2 | 3 | 4 | 5 |
| 1. …I experienced a warm feeling with the people I spent time with. | 1 | 2 | 3 | 4 | 5 |
| 1. …I felt insecure about my abilities. | 1 | 2 | 3 | 4 | 5 |

Scoring:

Autonomy satisfaction: items 10, 15 Autonomy frustration: items 13, 18

Relatedness satisfaction: items 16, 20 Relatedness frustration: items 12, 17

Competence satisfaction: items 14, 19 Competence frustration: items 11, 21

### **Psychological distress (GHQ-12)**

*We would like to know how healthy you feel at the moment and what complaints you have had in the past 7 days. Please note that these questions only concern the complaints of the present time or of the past 7 days and not complaints you may have had in the past.*

*The questionnaire consists of 12 items. Please indicate each time which answer is most applicable to you. By 'before' we mean the weeks before the COVID pandemic.*

1. Have you lost (much) sleep over worry over the past seven days?
   1. Not at all
   2. Not more than before
   3. A little more than before
   4. Much more than before
2. Have you felt (constantly) under strain over the past seven days?
   1. Not at all
   2. Not more than before
   3. A little more than before
   4. Much more than before
3. Have you been able to concentrate on your tasks over the past seven days?
   1. Better than before
   2. Just as well as before
   3. Worse than before
   4. Much worse than before
4. Have you felt that you were playing a useful part in things over the past seven days?
   1. More useful than before
   2. As useful as before
   3. Less useful than before
   4. Much less useful than before
5. Have you been able to face up to your problems over the past seven days?
   1. Better than before
   2. As well as before
   3. Less able than before
   4. Much less able than before
6. Have you felt capable to make decisions about things over the past seven days?
   1. More capable than before
   2. As capable as before
   3. Somewhat less capable than before
   4. Much less capable than before
7. Have you felt that you could not overcome your difficulties over the past seven days?
   1. No, I did not have that feeling at all
   2. Not less mastered than before
   3. A little less mastered than before
   4. Much less mastered than before
8. Have you been feeling reasonably happy over the past seven days, all things considered?
   1. Happier than before
   2. As happy as before
   3. Less happy than before
   4. Much less happy than before
9. Have you been able to enjoy your normal day-to-day activities over the past seven days?
   1. More than before
   2. As much as before
   3. A little less than before
   4. Much less than before
10. Have you been feeling unhappy or depressed over the past seven days?
    1. Not at all
    2. Not more than before
    3. A little more than before
    4. Much more than before
11. Have you been losing confidence in yourself over the past seven days?
    1. Not at all
    2. Not more than before
    3. A little more than before
    4. A lot more than before
12. Have you been thinking of yourself as a worthless person over the past seven days?
    1. Not at all
    2. Not more than before
    3. A little more than before
    4. Much more than before
