# Supplementary material for: Psychological distress among frontline workers during the COVID-19 pandemic: A mixed-methods study
Source: PLoS One. 2021 Aug 5;16(8):e0255510. doi: 10.1371/journal.pone.0255510 (PMC8341539; doi:10.1371/journal.pone.0255510)
Supplement: S2 File — Instructions and prompts for participants to record and share their entries. (DOCX) [file pone.0255510.s002.docx]

## **S2 File. Audio diary instructions.**

We would like you to make 2-3 audio-recordings per week – over a period of three weeks – about work-related experiences that had an impact on you. Please be discreet in your recordings and preferably make the recordings in a quiet place. You can record the audio-messages with your smartphone or another recording device. You can make recordings at any time of the day, both during and after a shift. Just keep in mind that you record the experiences as soon as possible after they took place, so that you still know as much detail as possible.

Please share your audio diaries within 24 hours after recording by e-mail, Telegram or WhatsApp with WEvdG. WEvdG will provide your recordings with a date and code and store them on a protected environment of the UMCG. Co-researchers and third parties have no access to this protected environment. Next, all audio diaries will be transcribed verbatim. All information that can be traced back to persons will be pseudonymised.

Notes on the content of the recording:

- Start the recording by providing your name and the date.
- Can you reflect on a work experience or situation (positive or negative) that made a big impact on you today? Please discuss this experience in as much detail as possible, including:
- In what context did this experience occur? (Situation)
- Who were involved? Do not record names, but name your relationship to these persons and the roles they fulfil. (Who)
- Why did you choose to share this experience?
- What insights has this experience brought you?
- Can you also explain in more detail in which way this situation affects you emotionally?

If applicable:

- What did you find difficult about this situation and what did you find easy?
- To what extent did you or did not feel competent to deal with this situation?
- To what extent did you feel or not feel sufficiently prepared to deal with this situation?
- To what extent did you feel supported by those involved?
- How did you experience the collaboration with those involved?

You may include all other aspects you deem important in this situation.

Please be aware that we do not expect you to spend any longer than 5 minutes per recording, unless you wish to.
