# Supplementary material for: Psychological distress among frontline workers during the COVID-19 pandemic: A mixed-methods study
Source: PLoS One. 2021 Aug 5;16(8):e0255510. doi: 10.1371/journal.pone.0255510 (PMC8341539; doi:10.1371/journal.pone.0255510)
Supplement: S3 File — The six steps of thematic analysis and the reflexivity paragraph. (DOCX) [file pone.0255510.s003.docx]

## **S3 File. Thematic analysis.**

We used a constructivist perspective to analyse the qualitative data, assuming that each participant shared their perspective on reality [33]. These different individual perspectives were used to interpret whether frontline workers’ basic psychological needs were satisfied or frustrated during COVID-19. To maintain confidentiality, all quotes only included participants’ respondent number. We used the six phases of thematic analysis to structure our analysis [34]. Data analysis started with familiarisation with the data by reading and re-reading all of the transcripts (WEvdG, RI, and KEN). Second, the transcripts were open-coded. The initial codes and memos about the coding process were discussed in weekly sessions (WEvdG, RI, and KEN). RI and KEN wrote a Master of Medicine’s thesis about the data. Third, WEvdG developed a deductive thematic framework with autonomy, competence, and relatedness satisfaction and frustration, respectively. Fourth, the analysis shifted to a deductive, latent thematic analysis, to code all transcripts. This process implies that analysis was firmly theory-driven, and we searched for features within the data that produced or gave meaning to participants' experiences. Throughout this process, WEvdG and RJD had regular meetings to discuss and review the themes. Fifth, we (re)defined and interpreted the themes, leading to the results section in this paper. Finally, WEvdG produced the draft of the results and triangulated the qualitative and quantitative data. WEvdG, RJD, ADCJ, and WFWB discussed the emerging results during several meetings. All authors reviewed and commented on the results.

The professional background of the authors shaped the interpretation of the results. For reflexivity purposes, we therefore describe the background of all authors. WEvdG is a PhD candidate, and is trained as a psychologist and educationalist and brought her knowledge of SDT to the analysis. KEN and RI are final-year medical students and had first-hand experience of volunteering during the COVID-19 pandemic. RJD is a senior researcher in health professions education and a psychiatry trainee, who delivered care to inpatient psychiatric patients during the pandemic, where he experienced working in PPE and dealing with uncertainty amongst healthcare workers. MACF is a senior researcher in health professions education and emergency medicine specialist; he has experience with supporting and preparing trainees and staff to cope with healthcare crises. NWVY is a professor in sport- and performance psychology, and is an expert in SDT and quantitative research methods. ADCJ is a professor in health professions education and brought extensive research experience in qualitative and quantitative research methods. WFWB, AJJL, JET, ROBG and EK are clinical researchers and consultants who were involved in COVID-19 hospital care, so they had personal experience with the impact of the pandemic on themselves and their colleagues.

**Table S3. Six phases of thematic analysis.**

| Steps | What was done |
| --- | --- |
| 1. Familiarisation with the data | - Transcription of audio diaries into transcripts.  - Reading all transcripts globally.  - Weekly discussions with RI, KEN, WEvdG, RJD.  - Monthly team meetings with the research team. |
| 2. Generating initial codes | - Open coding of the transcripts in weekly sessions.  - Open coding of 15 transcripts per week followed by a weekly meeting to discuss the codes (WEvdG, RI, KEN).  - Bi-weekly meetings with RJD. |
| 3. Searching for themes | - Development of a framework of analysis based on the BPNs of SDT.  - Weekly meetings (WEvdG, RI, KEN) and bi-weekly meetings with RJD to discuss the themes. |
| 4. Reviewing potential themes | - Discuss the codes that are grouped under each need.  - Discuss the codes that could be grouped under the basic needs.  - Develop additional themes to group these codes under. |
| 5. Defining and naming the themes | - Define which aspects of the transcripts describe each individual need.  - Analyse how different elements are connected. |
| 6. Producing the report | - Organise the results in need supportive and need hampering experiences. |
