# Supplementary material for: Psychological distress among frontline workers during the COVID-19 pandemic: A mixed-methods study
Source: PLoS One. 2021 Aug 5;16(8):e0255510. doi: 10.1371/journal.pone.0255510 (PMC8341539; doi:10.1371/journal.pone.0255510)
Supplement: S1 Table — (DOCX) [file pone.0255510.s004.docx]

## **S1 Table. Distribution of between- and within-person variance for the predictor variables (null models).**

| **Variable** | **Within-person variance** | | **Between-person variance** | |
| --- | --- | --- | --- | --- |
|  | **χ ^2^** | **ICC** | **χ ^2^** | **ICC** |
| **Psychological distress** | 109.74** | 73% | 79.82** | 27% |
| **Autonomy satisfaction** | 498.35** | 68% | 463.07** | 32% |
| **Competence satisfaction** | 422.40** | 85% | 413.42** | 15% |
| **Relatedness satisfaction** | 536.42** | 68% | 499.60** | 32% |
| **Autonomy frustration** | 537.32** | 82% | 525.29** | 18% |
| **Competence frustration** | 502.80* | 63% | 452.65* | 37% |
| **Relatedness frustration** | 409.55** | 70% | 376.97** | 30% |

**p* < .05; ***p* < .001. ICC = intraclass correlation.
