# Supplementary material for: Psychological distress among frontline workers during the COVID-19 pandemic: A mixed-methods study
Source: PLoS One. 2021 Aug 5;16(8):e0255510. doi: 10.1371/journal.pone.0255510 (PMC8341539; doi:10.1371/journal.pone.0255510)
Supplement: S2 Table — (DOCX) [file pone.0255510.s005.docx]

## **S2 Table. Non-parametric test for differences in psychological distress.**

| **Measurement** | **GHQ ≤ 1** | **GHQ > 1** | **Mann-Whitney U** | **Wilcoxon W** | **Z** | ***p*** |
| --- | --- | --- | --- | --- | --- | --- |
|  | **median** | **median** |  |  |  |  |
| **T1** | 1.67 | 2.08 | 56.50 | 434.50 | -4.49 | < .001 |
| **T2** | 1.58 | 1.92 | 41.50 | 569.50 | -4.21 | < .001 |
| **T3** | 1.67 | 1.92 | 47.00 | 482.00 | -4.07 | < .001 |
| **T4** | 1.67 | 2.08 | 31.50 | 527.50 | -4.22 | < .001 |
| **T5** | 1.67 | 2.08 | 28.50 | 434.50 | -4.50 | < .001 |
| **T6** | 1.58 | 2.04 | 30.00 | 240.00 | -4.62 | < .001 |

Z = z-test value, *p* = p-value.
