# Supplementary material for: Psychological distress among frontline workers during the COVID-19 pandemic: A mixed-methods study
Source: PLoS One. 2021 Aug 5;16(8):e0255510. doi: 10.1371/journal.pone.0255510 (PMC8341539; doi:10.1371/journal.pone.0255510)
Supplement: S3 Table — (DOCX) [file pone.0255510.s006.docx]

## **S3 Table. Fluctuations in psychological distress over time.**

| **Parameter** | **Model** | | | |
| --- | --- | --- | --- | --- |
|  | **b** | **SE** | **t(39)** | ***p*** |
| **Intercept** | 1.85 | 0.04 | 42.05 | < .001 |
| **T2** | -0.10 | 0.05 | -1.79 | .081 |
| **T3** | -0.07 | 0.05 | -1.34 | .188 |
| **T4** | -0.11 | 0.05 | -2.02 | .050 |
| **T5** | -0.05 | 0.05 | -0.83 | .410 |
| **T6** | 0.01 | 0.06 | 0.09 | .930 |
| **Explained variance (%)** | | | | |
| **Between-person level** | -1.12 | |  |  |
| **Within-person level** | 2.25 | |  |  |
| **2*log likelihood** | 72.30 | |  |  |
| **χ ^2^_change null model_ (5)** | 7.52 | |  | .185 |

b = unstandardised coefficient, SE = standard error, t(df) = t-test statistic with degrees of freedom within brackets, *p* = p-value.
